# LATER models of neural decision behavior in choice tasks

**DOI:** 10.3389/fnint.2014.00067

**Published:** 2014-08-22

**Authors:** Imran Noorani

**Affiliations:** Department of Neurosurgery, Wessex Neurological Centre, University Hospital SouthamptonSouthampton, UK

**Keywords:** saccades, decision, LATER, eye, latency, reaction time, neuron

## Abstract

Reaction time has been increasingly used over the last few decades to provide information on neural decision processes: it is a direct reflection of decision time. Saccades provide an excellent paradigm for this because many of them can be made in a very short time and the underlying neural pathways are relatively well-known. LATER (linear approach to threshold with ergodic rate) is a model originally devised to explain reaction time distributions in simple decision tasks. Recently, however it is being extended to increasingly more advanced tasks, including those with decision errors and those requiring voluntary control such as the antisaccade task and those where sequential decisions are required. The strength of this modeling approach lies in its detailed, quantitative predictions of behavior, yet LATER models still retain their conceptual simplicity that made LATER initially successful in explaining reaction times in simple decision tasks.

## Introduction

The question of how we choose one option over another has intrigued neuroscientists in the field of neural decision-making for many decades. Performing one action rather than another, say running toward a bus instead of walking, requires more than just sensory input and a motor output. The brain has to integrate sensory information and somehow make a decision on what is the “best” course of action based on this information, and subsequently to implement this decision as a motor response. It is now widely believed that the brain accumulates sensory information toward a threshold level, at which point the evidence has become convincing enough for a certain decision to be selected (Carpenter and Williams, 1995; Schall, 1995). Such an accumulator model approach to neural decision-making has been applied to many important features of decision behavior, yet there is still much to be explained.

Reaction time is regarded as an experimental “window” into decision processes. Reaction time, or latency, is composed of more than the simple sum of times of for sensory input and motor output, and it is this extra time that reflects the time taken for the brain to choose a response. This additional processing time can also be called neural procrastination: the brain is taking longer than it needs to if sensory input and motor output were all that is needed for a behavioral response. As sensory and motor times are relatively fixed, reaction time is therefore a useful indicator of decision time. In experimental paradigms, reaction time varies between one trial and the next, even if exactly the same experimental conditions are maintained.

Saccades are the rapid eye gaze shift movements which we make a great number of times every day. A saccade represents the output of a decision: a choice of where to look. They are especially useful for studying decision in the laboratory because they are quick so many of them can be produced in a short space of time, they have a clear sensory input (e.g., a visual stimulus), and their neuronal pathways are relatively well known. It is unsurprising, therefore, that saccades have been widely used to produce reaction time data to give us insight into decision mechanisms.

The neural control of saccades is mediated primarily by frontal and parietal cortical regions, basal ganglia and the superior colliculus. Although decision-making for generating saccades was thought to be cortical (Schall, 2001) more recent work suggests the superior colliculus may be involved in decision-making (Phongphanphanee et al., 2014) and a model of superior collicular activity can potentially explain express saccades (Trappenberg et al., 2001). Races between potential decisions have also been identified in the basal ganglia (Schmidt et al., 2013). The frontal eye fields (FEF) in the cortex have been particularly studied as regions for decision-making in eye movements (Pierrot-Deseilligny et al., 2003). Neurons in these regions project directly to brain stem structures containing ocular motor neurons so that they can directly influence the production of saccades (Segraves, 1992; Shinoda et al., 2011). Following target appearance, there is accumulation of movement-related activity in these regions toward a threshold, and once the threshold is reached a saccade is triggered (Hanes and Schall, 1996). The neuronal mechanisms for saccadic control have been reviewed in detail elsewhere (Pierrot-Deseilligny et al., 2004), and lesion studies in humans have been helpful in delineating such mechanisms (Pierrot-Deseilligny et al., 2002; Machado and Rafal, 2004; Ramat et al., 2007).

## The LATER model

**Figure 1:**
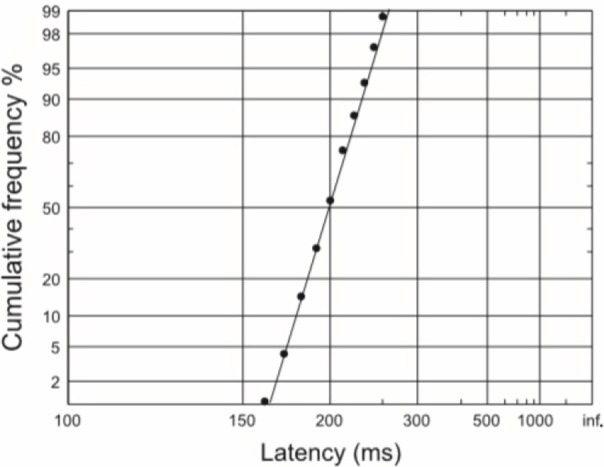
**A reciprobit plot of typical saccadic latencies, in which a straight line is obtained if reciprocal latencies are plotted on a cumulative y-axis scale**.

The variability of reaction times is an interesting phenomenon. When plotted on a histogram, the distribution of reaction times appears skewed. However, if we take the reciprocal of the latencies and plot these in a similar fashion, the resulting distribution appears Gaussian. This tells us that perhaps it is the reaction rate rather than reaction time *per se* which is a more important reflection of the underlying decision process. A Gaussian or normal distribution of reciprocal latencies implies that these reciprocals have equal variability around a mean value. If reciprocal latencies are plotted cumulatively (a reciprobit plot) then a straight line is obtained (Figure 1). Such a distribution can then be explained by a very simple and elegant model—the LATER model. It is important to note that although saccadic reaction times have been studied in great detail with this type of decision modeling, manual reaction times to auditory or visual stimuli have also been found to have straight line distributions much like saccades and can therefore be modeled similarly (Carpenter, 1981; Pearson and Carpenter, 2010).

The LATER (linear approach to threshold with ergodic rate) model is an established model of neural decision that has been highly successful in explaining reaction time distributions over the last few decades. In the model, a decision signal starts from a starting point S_0_ and rises toward a threshold ST—once the signal reaches the threshold, the decision is made for a particular action. The rate at which the decision signal rises varies randomly from trial to trial, but the mean rate of rise is constant and denoted by the parameter μ. The standard deviation of this variation in rate of rise is given by the parameter σ. One of the main reasons LATER is so conceptually attractive for explaining decisions is that the parameters of reciprocal reaction time distributions are the parameters of the model itself—μ being the mean rate of rise of the decision signal as well as the mean reciprocal latency, and σ reflecting the variability in the latency distributions (Carpenter and Williams, 1995). There are, of course, many other different kinds of accumulator race models which have been applied to various tasks; however, these have been discussed elsewhere including some attempts at comparisons between different types of model (Usher and McClelland, 2001; Ratcliff and Smith, 2004; Bogacz et al., 2006; Ratcliff and McKoon, 2008; Heathcote and Hayes, 2012; Heathcote and Love, 2012; Bitzer et al., 2014), and here we will focus on LATER.

**Figure 2:**
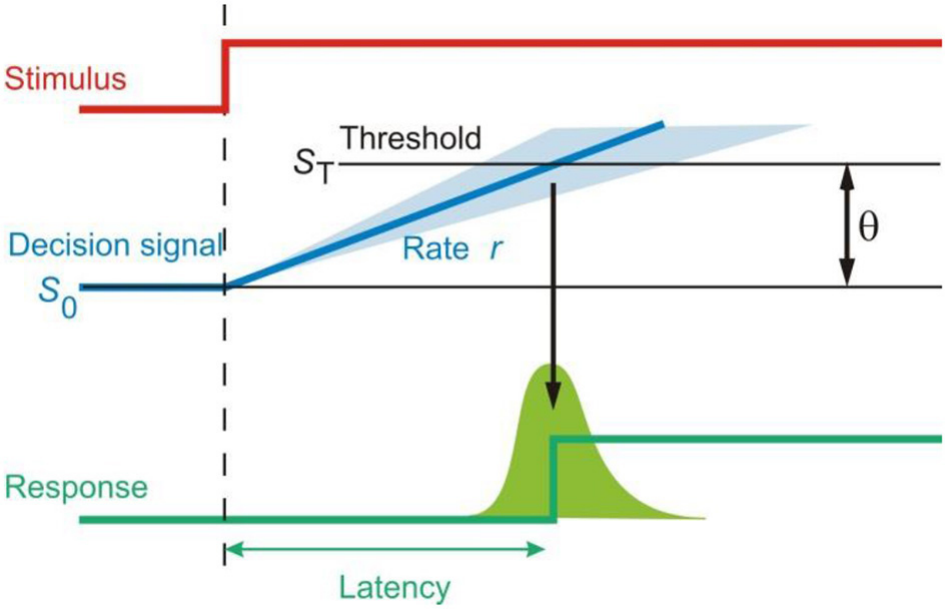
**The LATER model of decision**. A decision signal accumulates information about a decision based on information supply and rises toward threshold in a linear fashion, triggering a response when it reaches threshold.

What does this rise to threshold of the decision signal imply about the decision process? It is thought to be an accumulation of sensory “evidence” for a hypothesis, and this accumulation occurs in a linear fashion. Once enough evidence for a certain hypothesis is accumulated, signified by the decision signal reaching its threshold, then this hypothesis is accepted and a decision to respond is made (Figure 2). In this way, LATER is a quasi-Bayesian model of decision-making and the decision signal itself mathematically corresponds to the log likelihood ratio of a certain choice being the correct one (Carpenter and Williams, 1995). One can easily therefore conceive many possible choices, and the final choice out of a number of options occurs when the decision signal for this choice reaches threshold before the decision signals representing the other options. Indeed, a simple task with a visual target and a visual distractor was modeled by two LATER units, one representing the target and the other the distractor, both racing against each other to threshold to determine the response (Leach and Carpenter, 2001).

Any robust neural model must be subject to empirically testable predictions, and the LATER model is no exception. When reaction time distributions are plotted on a reciprobit plot, a straight line is obtained if the reciprocal latencies follow a Gaussian distribution. If the mean rate of rise of the decision signal is increased, then one would predict that the latency distribution should shift toward shorter latencies. If we alter S_0_ by varying prior probability of a stimulus, then one would predict a swivel in the latency distribution. Indeed, these predictions were borne out in an elegant set of experiments. The mean rate of rise was altered by changing the rate of information provision and prior probability was altered by changing subject's expectations of stimulus locations—both manipulations produced the changes in latency distributions predicted by the LATER model (Carpenter and Williams, 1995; Reddi and Carpenter, 2000; Reddi et al., 2003). The importance of predicting complete distributions of reaction times as opposed to mean/median latencies or latency quantiles lies in the fact that there can be subtleties to a full distribution that often point to multiple decision mechanisms and these could be missed if the distribution is not plotted in full (Noorani and Carpenter, 2011).

LATER is a model of decision processes. Reaction time however is composed of sensory detection and motor implementation, as well as decision. When the visual targets are high-contrast and thus easily detectable, a LATERian approach predicts reaction times very well; but when the targets are more difficult to detect, a random-walk model that integrates noisy afferent signals models behavior well. Naturally, the models can be reconciled by viewing stimulus detection and decision as two separate processes occurring sequentially, with stimulus detection occurring in a random-walk fashion followed by the decision process occurring in a LATERian manner. A model of this sort predicts reaction time distributions over a wide range of stimulus detectability (Carpenter et al., 2009).

## Early saccades

**Figure 3:**
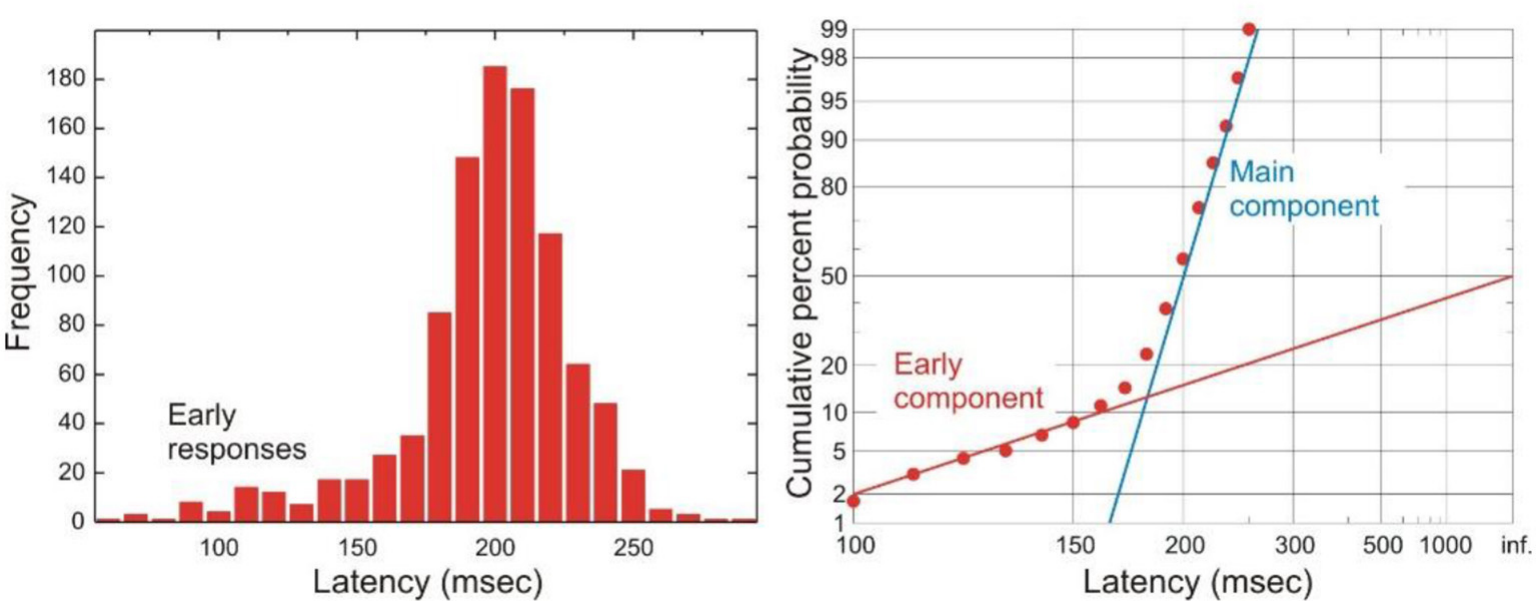
**A typical histogram of latencies is shown on the left, and a reciprobit plot of the same data on the right**. A prominent component of early saccades is seen in this distribution. Modified with permission from Noorani and Carpenter (2011).

On a small number of occasions, experimental subjects produce saccades whose latencies are very short and do not follow the main latency distribution. Such “early” responses form a separate component of the latency distribution on a reciprobit plot, as a straight line with a shallower gradient than the main component of the distribution (Figure 3). These early responses therefore cannot be explained by the same single LATER decision unit that gives rise to saccades that follow the main distribution (otherwise they would be part the same straight line on the reciprobit). Instead, it has been proposed that these early responses are the result of an “early” LATER decision unit, whose parameters differ from those of the main LATER unit in having a mean rate of rise of zero but a very large σ, such that occasionally this eccentric unit wins the race against the main unit to produce a fast response. Simulations with such a race between these two decision units do indeed produce latency distributions with an early and main component mirroring real life behavior (Noorani and Carpenter, 2011). One circumstance in which the frequency of early responses is increased is under conditions of cognitive distraction. This has been demonstrated experimentally when a subject is using a mobile phone whilst performing a simple saccadic task, in which there is a larger early component to the latency distribution. Perhaps when one is distracted there is less cortical inhibition from “higher” regions like supplementary and frontal eye fields to more primitive neural regions controlling saccades, such as the superior colliculus, allowing these maverick early responses to win the decision race more often (Halliday and Carpenter, 2010).

## Countermanding and gap paradigms

**Figure 4:**
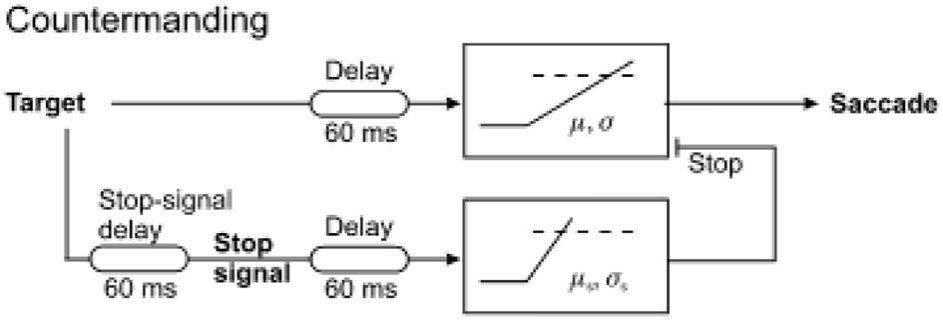
**The countermanding race model, in which a stop unit competes with a go unit and the winner of the race determines the outcome**. Trials in which responses failed to be canceled despite presence of a stop signal are accounted for by the go unit reaching threshold before stop. Modified with permission from Noorani et al. (2011).

The situation frequently arises when one needs to cancel an impending an action, for example stopping yourself from crossing the street as a car suddenly drives past. In scenarios like this, there must be a way of suppressing a decision signal that is itself accumulating and about to generate a response. An established paradigm for studying this behavior in the laboratory is called the countermanding task. Here, a visual stimulus appears and the subject knows they must make a saccade toward it, but sometimes after the target appears a “stop” signal also appears indicating to the subject that they must not make a saccade to the target. On these “stop” trials, the developing decision to generate a saccade must be canceled. This is called the “countermanding” task. What is the underlying neural mechanism enabling this to be achieved? It requires the involution of a new concept—the stop unit, a decision signal which can accumulate evidence needed for canceling a response when a stop signal is presented. The stop unit must race against the “go” unit, which is responsible for triggering a saccade (Figure 4). In this way, on some trials no saccade occurs because the stop unit wins the race, whereas on others a saccade occurs to the stimulus when the go unit reaches threshold first (Logan et al., 1984). Hanes and Carpenter then demonstrated that a linear rise to threshold for stop and go processes can successfully account for the detailed reaction time distributions in this task, with the mean and variability of the rate of rise of the two types of unit will determine the precise timing and frequency of these two types of response. Such a simple model can robustly predict not just the mean latencies but also (and much more importantly) the latency distributions and incidence of stop and go responses (Hanes and Schall, 1995; Hanes and Carpenter, 1999; Boucher et al., 2007). It must be noted, however, that recent work suggests that stimulus detection or perception may play a larger role in countermanding than previous considered (Salinas and Stanford, 2013). Moreover, countermanding is distinct from task-switching in which a new instruction puts the old one out of date: in this case, this is likely to occur through a functional unit to detect the new instruction which then activates a separate LATER unit to accumulate activity for the appropriate decision (Sinha et al., 2006).

Another interesting paradigm is the gap task, in which a central fixation unit disappears to leave a short period in which there is no visual stimulus before a peripheral stimulus appears. This “gap” in between stimuli has been found to speed up reaction times, perhaps because it provides a warning effect signaling to the subject the impending appearance of a stimulus. This has also been modeled by two racing LATER units, except unlike in countermanding there is no stop unit but instead there is a “fixation” unit in addition to the main saccadic unit. The fixation unit is activated when the fixation stimulus disappears, and instead of stopping the main saccadic unit it *enhances* its decision signal allowing it to reach threshold more quickly. In this way, this model quantitatively predicts the reaction time distributions in the gap paradigm (Story and Carpenter, 2009).

## Go/no-go paradigm

From this early work, LATER gained substantial popularity as a model of decision-making because of its success in explaining full reaction time distributions in simple decision tasks in such a simple conceptual manner. However, no model of decision would be complete without being able to explain how wrong decisions come about: people often make errors in their choices. To address this important problem, we needed a task that would induce subjects to make a large number of errors, such that the latencies of these responses could be studied in detail to enable us to gain insight into the underlying decision process. The go/no-go task is an experimental paradigm in which a visual stimulus is presented that signifies the subject to make a saccade toward it, but on some occasions a different stimulus is presented that the subject has been pre-warned not to respond to. Although the instructions are clear to the subject, they still respond to the latter stimulus sometimes—this is classed as an error, a wrong decision. How can we make this task produce many errors? We did this by making the two types of visual stimuli with the same shape and size, but of different color—a red dot and a blue dot, one of which is “correct” and one is an “error.” In this way, color is the only distinguishing feature of the two stimuli, making it rather easy for a subject to make an error by simply responding to a novel stimulus of the “wrong” color (Noorani et al., 2011).

**Figure 5:**
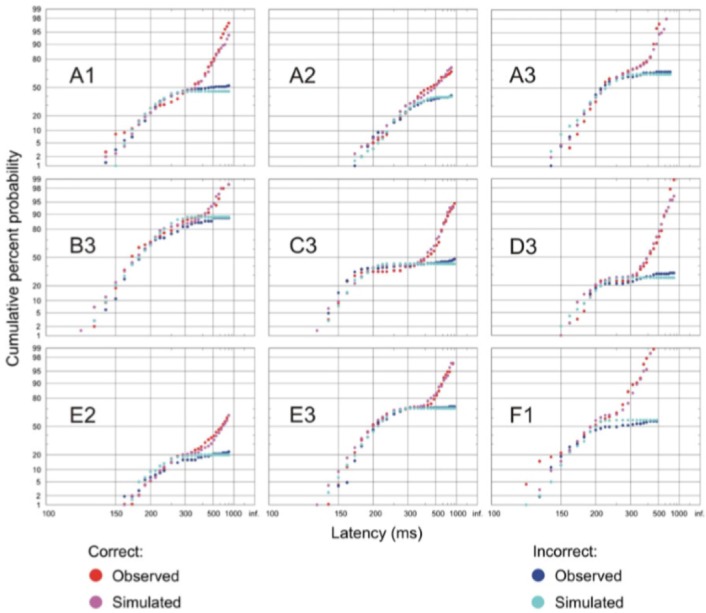
**Go/no-go task reaction time distributions of correct and error responses**. Initially, the correct and error distributions overlap, then the correct distribution rises further whilst the error one tends to flatten at longer latencies. Simulations match the observed distributions in all cases (*p* < 0.05, Kolmogorov–Smirnov test). Modified with permission from Noorani et al. (2011).

**Figure 6:**
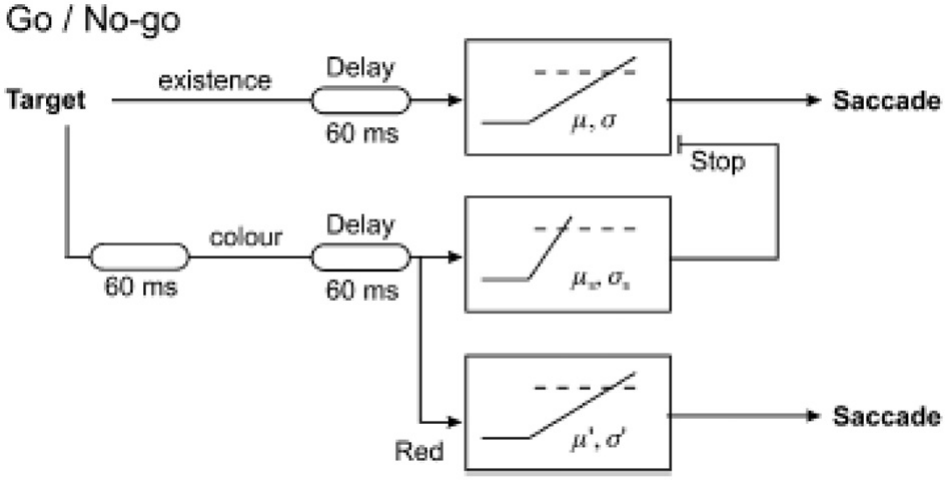
**LATERian model of the go/no-go task, in which there are three competing units**. The existence unit is activated by any visual stimulus regardless of color, generally producing faster responses. The color unit is activated when a stimulus of the correct color appears, but there is an extra 60 ms delay before it is activated representing the time for color information to arrive in cortex. The stop unit is similarly activated at the same time to cancel the impending non-selective response from the existence unit. Modified with permission from Noorani et al. (2011).

The resulting latency distributions from this task are rather distinctive (Figure 5). If the error response and correct response distributions are plotted separately on a reciprobit plot, the two distributions initially overlap, but after a further 60 ms or so, the two distributions begin to diverge with the error distribution starting to flatten off whilst the correct distribution rises further. This can be explained by the arrival of color information in the cortex after this extra delay, allowing the cortex to make a correct decision of whether to respond based on the color of the stimulus. Before this time, such information is not available to the cortex to make an informed decision and therefore the probability of making a correct response is the same as that of an error. A LATERian approach to modeling these data takes us back to the countermanding model, wherein the stop unit is of primary importance. The initial part of both correct and error distributions is modeled by an “existence” unit which rises toward threshold on presentation of any visual stimulus, regardless of its color. If this reaches threshold, a saccade will occur toward the stimulus, and this can therefore be a correct or an error response depending on the color of the stimulus. It will typically be a quick response, as the unit accumulates immediately on appearance of a stimulus. However, after another 60 ms delay when color information arrives in cortex (Thompson et al., 1996; Schall and Bichot, 1998), a separate “color” LATER unit and a stop unit activate. The stop unit has a fast mean rate of rise, enabling it to frequently cancel the existence unit (assuming it has not yet reached threshold), whilst the color unit will produce a correct saccade if it wins the race (Figure 6). This explains why the correct latency distribution in the go/no-go task has two steep components: the first one being generated by the existence unit, and the later part being produced by the color unit. The stop unit ensures few errors are made after color information arrives; hence the error distribution flattens off.

## The anti-saccade task

**Figure 7:**
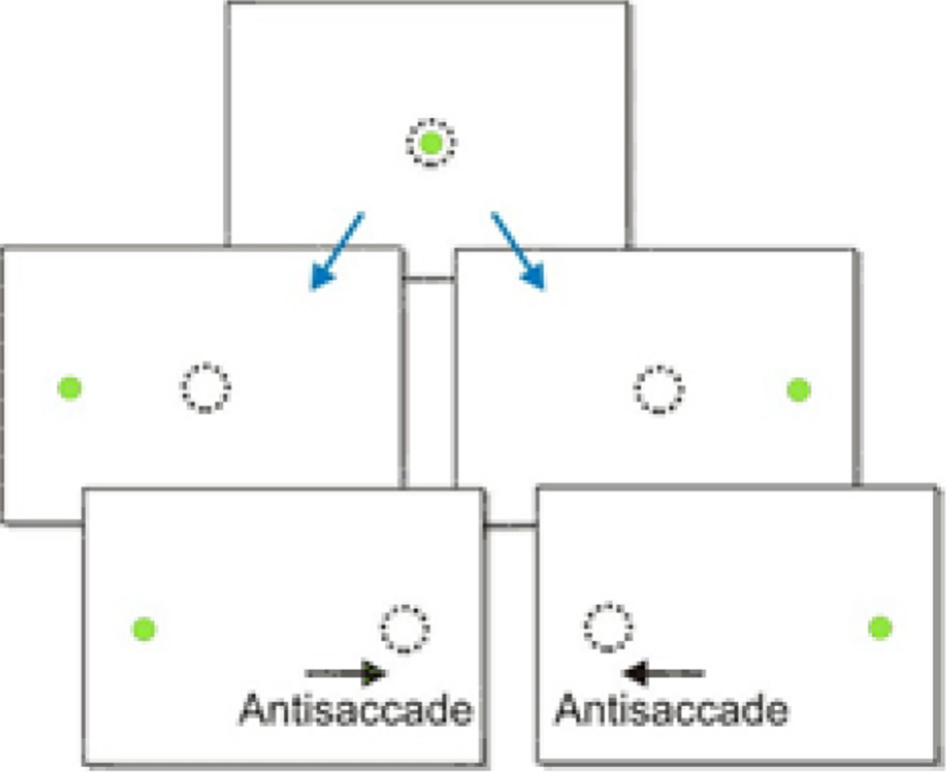
**The antisaccade paradigm**. A subject is told to look in the opposite direction to the presented stimulus.

Another and perhaps more complex kind of saccadic decision task is the anti-saccade task. Anti-saccades are saccadic eye movements in the opposite direction to a visual stimulus. This is a much more challenging response than a typical saccade because it requires a subject to withhold a saccade to a novel stimulus and instead look away from it (Figure 7). Consequently, anti-saccades are often used as an experimental paradigm for studying behavioral control, and are increasingly being examined in clinical conditions such as Parkinson's disease and Alzheimer's disease as a marker of voluntary control (Munoz and Everling, 2004). Anti-saccades are typically slower than normal saccades, and in this task a pro-saccade to the visual stimulus is an error (Fischer and Weber, 1992).

**Figure 8:**
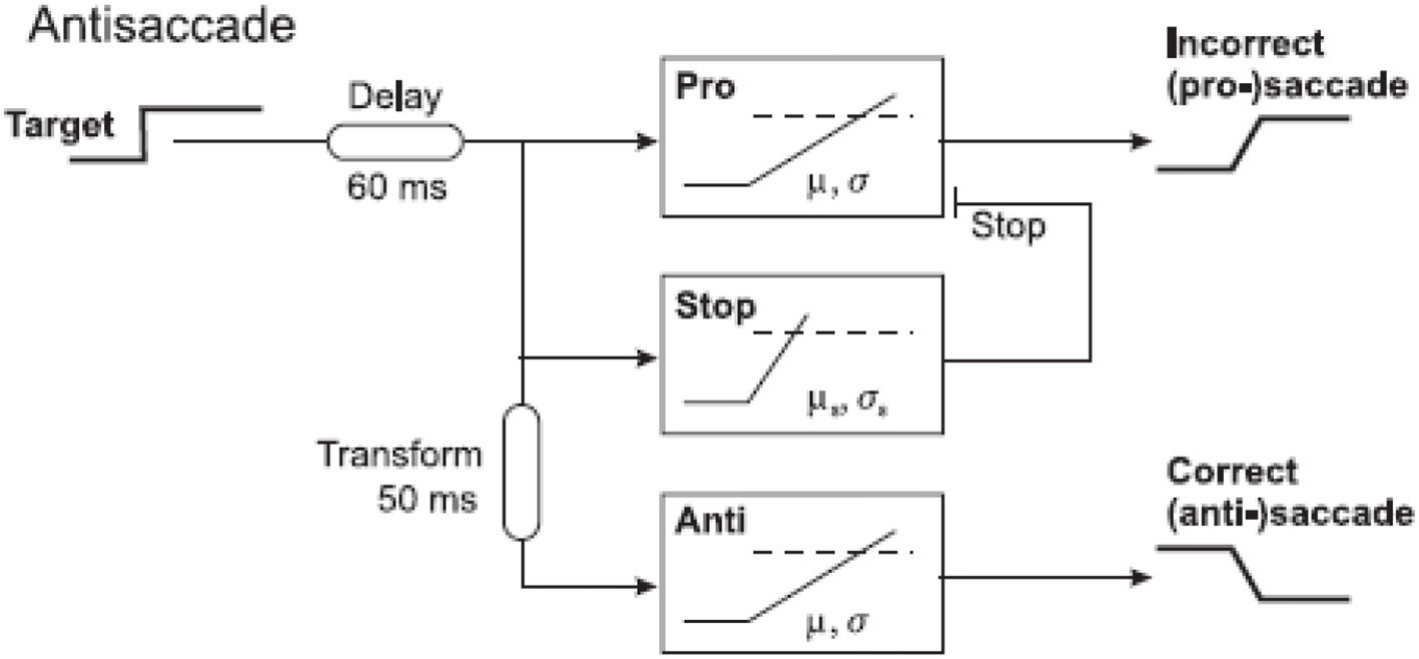
**Three-unit antisaccade model**. The pro-saccade unit is turned on by appearance of a stimulus. After a further 50 ms (time taken for vector inversion in the lateral intraparietal area), the stop and antisaccade units are activated. This model can therefore produce errors and antisaccades.

**Figure 9:**
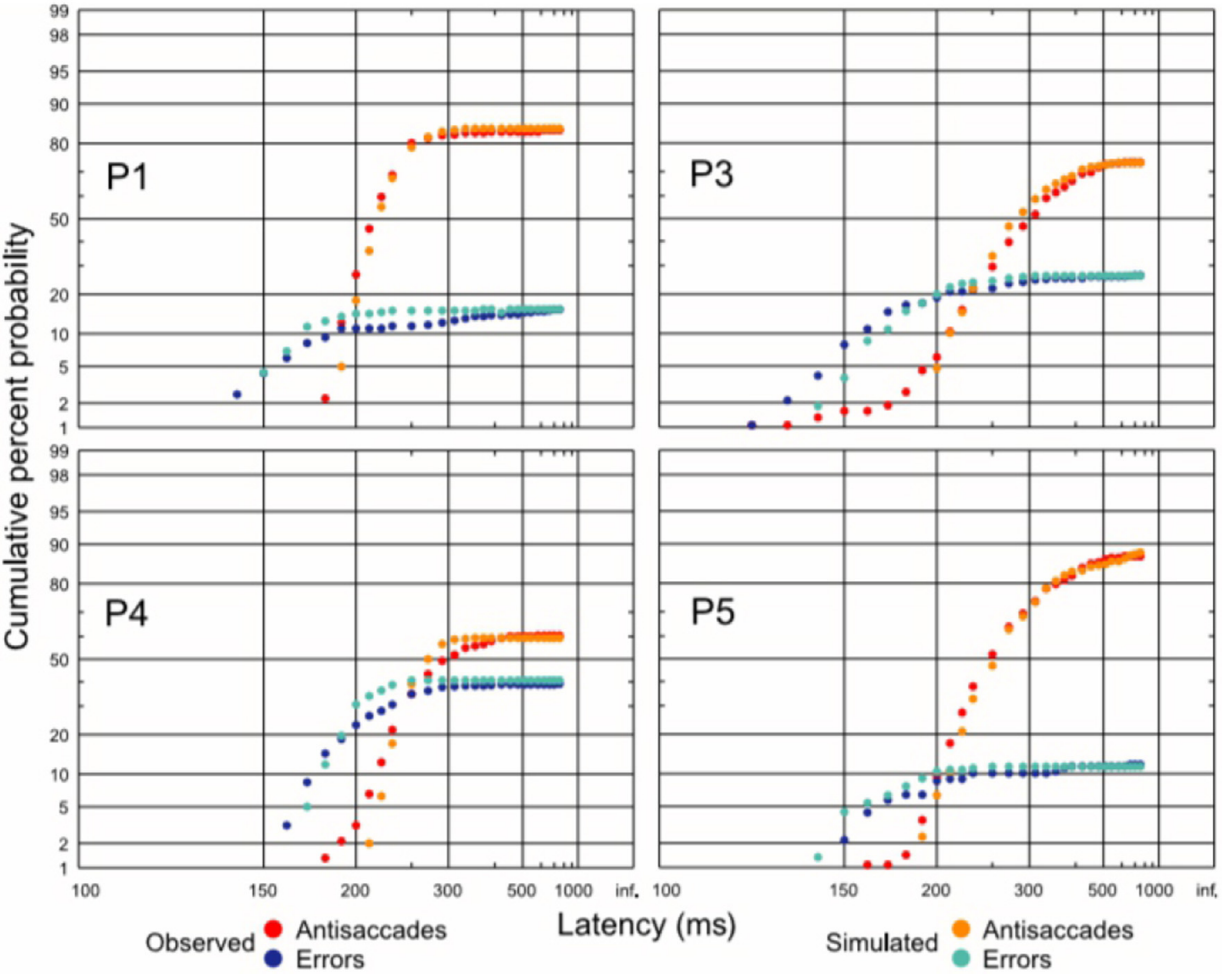
**Antisaccade and error distributions (observed and predicted), demonstrating that the three unit-unit LATER model for antisaccades can quantitatively predict reaction time distributions in this complex task**. Modified with permission from Noorani and Carpenter (2011).

Given their wide relevance, it is important to understand the neural processes by which anti-saccades come about. A study in monkeys using neuronal recordings demonstrated that a vector inversion is calculated in the lateral intraparietal area after a 50 ms delay, a necessary prerequisite for producing a movement in the opposite direction to a stimulus (Gottlieb and Goldberg, 1999; Zhang and Barash, 2000). Using a similar approach as for the go/no-go task, our laboratory asked subjects to perform the anti-saccade task and we plotted their data as separate anti-saccade and error distributions on a reciprobit plot. The distributions for this task are unique: the error distribution begins earlier than the anti-saccade one and levels off earlier too. The error rate varies greatly from subject to subject but typically is around 5–30%. A LATER modeling approach demonstrated that a three-unit model fitted the data most accurately with the fewest free parameters, and this was composed of an error unit that responds to the sudden appearance of a novel stimulus, a stop unit which acts to cancel the developing error response, and the anti-saccade unit which begins accumulating after a vector inversion delay of 50 ms (Noorani and Carpenter, 2013; Figures 8, 9).

To perform an even more stringent test of this model, subjects were asked to perform the task under varying conditions of prior probability as this is known to affect the distributions (Koval et al., 2004). For example, in one version of the task there was an 80% chance of the stimulus appearing on the left and a 20% chance of it appearing on the right. These conditions created large shifts in the latency distributions of both correct and error responses and also greatly affected the error rates. Just as for a simple saccadic task wherein prior probability is explained by changes in the distance to threshold of a LATER unit, it was hypothesized that this alteration of the appropriate LATER units would account for the changes in the observed latency distributions of the anti-saccade task under varying conditions of subject expectation. Indeed this was the case: altering the distance to threshold of the decision units accurately predicted the anti-saccade and error distributions with different prior probabilities. Previous models of antisaccades have not incorporated a stop unit (Kristjansson et al., 2001; Cutsuridis et al., 2007). Crucially, alterations in the stop unit's distance to threshold were necessary to predict the large changes in error rates when prior probability is altered, highlighting the importance of the stop unit in this voluntary behavioral task (Noorani and Carpenter, 2013).

A widely recognized observation in the antisaccade task is the correctional eye movements that occur following an error: subjects often tend to make an anti-saccade after they make an error saccade in order to correct their mistakes (Mokler and Fischer, 1999). Corrections generally occur after the vast majority of errors in the anti-saccade task, and they are typically quick responses. However, how such corrections would be generated from a neural race model is not immediately obvious since the first response in the task (error or antisaccade) is the result of a decision unit having won the race and implying the race has ended. How can another decision be made after the race has already finished? In order to answer this question, the latency distributions of these separate responses had to first be analyzed in detail. A separate study was designed to record these new correction responses, in addition to errors and anti-saccades, allowing the three distributions to be plotted separately. The correction distribution was seen to be shifted to later time points compared to errors and anti-saccades, with a frequency typically slightly less than errors (after all, most errors are corrected). Two possibilities could potentially explain the correction distribution from the basic anti-saccade model:

1. Race continues after the error unit wins. If the error unit wins and an error is thus produced, a correction can ensue if the race is allowed continue thereby allowing the accumulating anti-saccade unit to carry on rising toward threshold and generate an anti-saccade. Although this is a plausible solution, this model predicts only fast corrections, so is not able to capture the whole reaction time distribution for corrections.
2. Race re-starts after the error unit wins. This is a novel concept for race models. Instead of the race completely ending when an error has been made, the error unit finishes the race as expected but then the antisaccade unit re-starts from scratch. It as if the brain knows an error has been made and in order to correct the mistake it re-sets the antisaccade unit, this time with no competition from the error unit. Using the same parameters as for the basic anti-saccade model, this new model accurately predicted all latency distributions in detail, including that of the correction responses (Noorani and Carpenter, 2014).

In order to re-start a race after a decision unit has won, there must be a way of monitoring the outcome of decision races. Such an idea is not itself a new one, for example there is some evidence that the supplementary eye field monitors the results of decision processes regarding eye movements (Carpenter, 2004) and this area is thought to be important in anti-saccades (Chapman and Corneil, 2014).

## Reaction time as a clinical biomarker

Saccades are increasingly being explored as potential novel biomarkers of neurological and psychiatric diseases, in particular for improving diagnostic accuracy and monitoring disease progression (Leigh and Kennard, 2004). For example, abnormalities in saccadic latency have been found in Parkinson's disease, and these patients also have higher error rates in the anti-saccade task (Anderson and MacAskill, 2013). Anti-saccade deficits have been found in patients with Huntington's disease too (Peltsch et al., 2008). LATER models of the basic saccadic step task and the increasingly complex types of saccadic task promise to be helpful in improving analysis and interpretation of eye movement deficits in such neuropsychiatric diseases (Antoniades et al., 2010). The main advantage of the models presented in this review is that they have been grounded in detailed quantitative predictions of full reaction time distributions: a very stringent test of any decision model. This promises to be useful when applied to reaction time distributions to patients with these conditions, in which mean latencies and other task parameters are abnormal, as it will allow correlation of task deficits with model parameters. Indeed, a recent international consensus has been reached regarding an optimal protocol of the anti-saccade task for use in clinical research (Antoniades et al., 2013).

## Conclusions and future directions

The use of saccades in neural decision research has proved a very useful approach. In particular, simulating complete reaction time distributions has been a triumph of the LATER models of decision, which in recent years has been applied to increasingly complex tasks including the go/no-go and antisaccade tasks. Given their simplicity, LATER models have proved useful for conceptualizing advanced decision processes. Clinical research is beginning to incorporate saccadic latency as a potential biomarker for neuropsychiatric disease. Recent research has excitingly begun to provide insight to how decisions with multiple options are generated, with evidence suggesting that this may involve a race between different neural pathways (Schmidt et al., 2013), rather like the way in which the LATER model has been applied to explain behavioral data in such decision tasks. Emerging work is also beginning to directly link neuronal activity with parameters of accumulator decision models (Purcell et al., 2010, 2012). Challenges of the future will be to correlate LATERian predictions with neuronal activity by direct neuronal recordings in order to demonstrate where and how such decision processes occur in the brain. We have seen how complex tasks can be modeled with multiple LATER units representing different possible response options, and it is likely that these units can be correlated with groups of neurons in the known oculomotor regions whose activity represent developing decisions.

### Conflict of interest statement

The author declares that the research was conducted in the absence of any commercial or financial relationships that could be construed as a potential conflict of interest.
